# Diagnostic Value of the Terminal D1S + D3R Pattern for Detecting Right Ventricular Dilatation in Patients with Atrial Septal Defect

**DOI:** 10.3390/jcdd13060245

**Published:** 2026-06-03

**Authors:** Rauf Avcı, Fatih Han Kumtaş

**Affiliations:** 1 Turkish Ministry of Health Antalya Training and Research Hospital, 07100 Antalya, Turkey; 2 Cardiology Department, Elbistan State Hospital, 46300 Kahramanmaraş, Turkey; fthhn16@gmail.com

**Keywords:** atrial septal defect, 12-lead electrocardiogram, terminal D1S + D3R, right ventricular dilatation

## Abstract

Background: Atrial septal defect (ASD) is common in adults and may cause chronic right ventricular (RV) volume overload and remodeling. Electrocardiography (ECG) may serve as a screening adjunct to echocardiography. Objectives: To evaluate the association of the terminal D1S + D3R ECG pattern, defined as a terminal S wave in lead I plus a terminal R wave in lead III, with structural and hemodynamic right heart involvement in adult secundum ASD. Methods: A total of 161 adult patients with secundum ASD were retrospectively analyzed. Right heart involvement was assessed using pulmonary-to-systemic flow ratio (Qp/Qs) ≥ 1.5 and a right ventricular/left ventricular (RV/LV) ratio > 1. ECG parameters, including right bundle branch block (RBBB), right axis deviation, V1–V2 R-wave positivity, and terminal D1S + D3R, were evaluated by two blinded cardiologists, with final classifications determined by consensus. Multivariable Firth penalized logistic regression, correlation analyses, and receiver operating characteristic (ROC) analyses were performed. Results: In the multivariable Firth penalized logistic regression model, pulmonary artery pressure (PAP) and ASD diameter were independently associated with Qp/Qs ≥ 1.5, whereas the terminal D1S + D3R pattern was not. The terminal D1S + D3R pattern was independently associated with RV dilatation after adjustment for age, sex, PAP, and ASD diameter (odds ratio [OR]: 9.90, 95% confidence interval [CI]: 2.82–38.20, *p* < 0.001) and showed good discriminatory performance for RV dilatation (area under the curve [AUC]: 0.881, 95% CI: 0.831–0.932). Conclusions: In adults with secundum ASD, a positive terminal D1S + D3R ECG pattern is independently associated with RV dilatation and may serve as a practical adjunctive screening marker. However, it should not replace echocardiographic assessment.

## 1. Introduction

Atrial septal defect (ASD) is one of the most common congenital heart diseases (CHD) observed in adults [1,2]. Secundum ASD accounts for approximately 70% of all cases [1,2]. Although many patients remain asymptomatic during childhood, chronic right ventricular (RV) volume overload may eventually lead to RV dilatation, right heart failure, arrhythmias, and pulmonary arterial hypertension (PAH) [3].

Patients diagnosed with CHD are recommended to undergo echocardiographic evaluation for the development of PAH [4]. However, definitive diagnosis often requires right heart catheterization (RHC) [4,5]. Nevertheless, in some clinical settings, access to timely echocardiographic assessment may be delayed. Therefore, simple, inexpensive, and widely available tools such as electrocardiography (ECG) may serve as screening adjuncts that complement echocardiographic evaluation and increase clinical awareness of possible right heart involvement in patients with ASD.

The terminal D1S + D3R pattern is defined as a rightward and inferior shift in the terminal QRS vector, characterized by a terminal S wave in lead I and a terminal R wave in lead III. This pattern is associated with ECG terminal vector changes consistent with delayed right ventricular activation, likely secondary to chronic RV volume loading and structural remodeling. Compared with conventional ECG findings such as right bundle branch block (RBBB) or right axis deviation, the terminal D1S + D3R pattern may provide a practical surface ECG marker associated with right-sided structural involvement in patients with secundum ASD. However, no prior adult ASD study has specifically evaluated the terminal-phase D1S + D3R vector shift as an ECG marker associated with RV structural remodeling.

This study aimed to evaluate the association of the terminal D1S + D3R pattern with structural and hemodynamic indicators of right heart involvement, with particular emphasis on RV dilatation.

## 2. Materials and Methods

### 2.1. The Study Design and Patient Population

Patients diagnosed with ASD at the Antalya Training and Research Hospital between January 2020 and June 2025 were retrospectively reviewed for inclusion. Patient data, including ECG, echocardiography, transesophageal echocardiography (TEE), and laboratory parameters, were obtained from the hospital electronic records. ASD diameters were assessed using TEE, and Pulmonary-to-Systemic Flow Ratio (Qp/Qs) was determined by transthoracic echocardiography.

In patients who underwent percutaneous ASD closure, echocardiographic parameters used for analysis were derived from transthoracic and/or transesophageal echocardiographic examinations performed prior to the intervention. As routine right heart catheterization is not performed before ASD closure at our center, invasive hemodynamic data were not available in this study.

Patients aged >18 years with a diagnosis of secundum ASD were included. Individuals with interstitial lung disease, primary PAH, moderate/severe left-sided valvular disease, left ventricular ejection fraction < 60%, pacemakers, or low-quality ECGs were excluded.

RV dilatation was defined as a right ventricle/left ventricle (RV/LV) ratio > 1, calculated from basal RV and LV diameters reported in routine echocardiographic assessments [6]. Right ventricular load was considered to consist of two distinct physiological components: hemodynamic burden and structural enlargement. While a Qp/Qs ratio ≥ 1.5 represents hemodynamic overload associated with shunt volume, an RV/LV ratio > 1 reflects structural remodeling of the RV. Therefore, recognizing that these two indicators are not interchangeable, separate sub-analyses were performed to individually evaluate structural (RV/LV) and hemodynamic (Qp/Qs) parameters in order to strengthen the validity of the predictive assessments.

Qp/Qs is calculated by transthoracic echocardiography using Doppler-derived stroke volume measurements. Qp is obtained from the right ventricular outflow tract (RVOT) at the pulmonic valve level, while Qs is obtained from the left ventricular outflow tract (LVOT) at the aortic valve level. For each outflow tract, the cross-sectional area is calculated from the measured diameter using the formula CSA = π × (diameter/2)^2^. Stroke volume is calculated as SV = CSA × velocity–time integral (VTI). Accordingly, the Qp/Qs ratio is calculated using the formula (CSA_RVOT × VTI_RVOT)/(CSA_LVOT × VTI_LVOT). Measurements are averaged over at least three consecutive cardiac cycles in sinus rhythm; in the presence of atrial fibrillation, a greater number of cycles is averaged.

**Figure 1:**
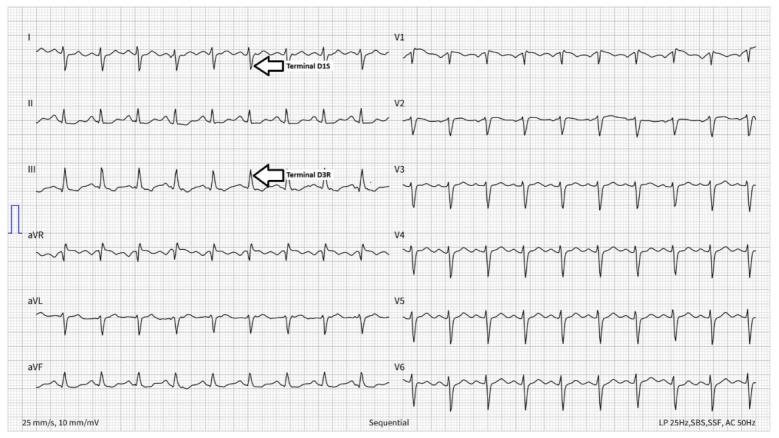
Terminal D1S + D3R Pattern in electrocardiography.

ECG parameters including RBBB, R-wave positivity in V1–V2, right axis deviation, and terminal D1S + D3R were evaluated for their association with RV dilatation. Terminal D1S was defined as a negative deflection with an amplitude ≥0.1 mV within the final 40 ms of the QRS complex in lead I. Terminal D3R was defined as a positive deflection with an amplitude ≥0.1 mV within the same time interval in lead III. The D1S + D3R pattern was considered positive only when both components were present simultaneously. Cases in which either a terminal S wave in lead I or a terminal R wave in lead III was observed alone were regarded as discordant or incomplete patterns and were classified as negative in the analyses. All measurements were performed manually at a paper speed of 25 mm/s and a calibration of 10 mm/mV. Figure 1 illustrates a representative electrocardiogram demonstrating terminal D1S and D3R patterns. All ECG parameters were evaluated by two cardiologists who were blinded to clinical data and echocardiographic findings. The final classification of each ECG parameter was determined by consensus assessment.

Right bundle branch block (RBBB) was defined according to standard 12-lead electrocardiographic criteria, including a QRS duration ≥ 120 ms, an rsR′, rSR′, or broad R-wave pattern in leads V1–V2, and a broad S wave in the lateral leads (I, V5–V6). Incomplete RBBB (iRBBB) was defined as the presence of an RBBB-like morphology in leads V1–V2 with a QRS duration of 110–119 ms.

Pulmonary artery pressure (PAP) refers to systolic pulmonary artery pressure, estimated from the tricuspid regurgitation (TR) jet velocity using the modified Bernoulli equation, and was analyzed as a continuous variable. PAH was not evaluated as a separate diagnostic entity or outcome in this study. Accordingly, all electrocardiographic and echocardiographic parameters were defined a priori using standard criteria.

Right and left ventricular dimensions were obtained from routine clinical echocardiography reports. In routine echocardiographic assessment at our center, RV and LV dimensions are evaluated from the apical four-chamber view at end-diastole, in accordance with the American Society of Echocardiography/European Association of Cardiovascular Imaging (ASE/EACVI) chamber quantification recommendations [6]. In the available reports, RV diameter referred to the basal right ventricular diameter, and LV diameter referred to the basal left ventricular diameter. The RV/LV ratio was calculated by dividing the basal RV diameter by the basal LV diameter. An RV/LV ratio > 1 was considered consistent with RV dilatation [6].

### 2.2. Statistical Analysis

All analyses were performed using IBM SPSS Statistics for Windows, Version 27.0 (IBM Corp., Armonk, NY, USA). The assumption of normal distribution for continuous variables was examined with the Kolmogorov–Smirnov and Shapiro–Wilk tests. The Mann–Whitney U test was applied to compare two independent groups that did not show a normal distribution. The Chi-square test was used to evaluate the relationship between categorical variables.

Correlation analyses were performed to evaluate the relationships among physiologically related variables, including PAP, ASD diameter, RV dilatation, and TR grade. Pearson or Spearman correlation coefficients were used for continuous variables according to distributional characteristics, whereas point-biserial or rank-biserial correlation coefficients were used for associations involving binary or ordinal variables. Multicollinearity among predictors included in the Firth-penalized logistic regression models was additionally evaluated using variance inflation factor and tolerance values.

To reduce the risk of overfitting, multivariable Firth penalized logistic regression models were constructed using five clinically relevant core variables: age, sex, PAP, ASD diameter, and the terminal D1S + D3R pattern. Firth penalized logistic regression was preferred to reduce potential sparse data bias and quasi-complete separation. Events-per-variable (EPV) values were calculated for each model to assess the adequacy of outcome events relative to the number of predictors. Model calibration was evaluated using the Hosmer–Lemeshow goodness-of-fit test.

The diagnostic performance of individual ECG parameters was evaluated using receiver operating characteristic curve analysis (ROC). For diagnostic performance analyses, each ECG parameter was treated as a binary variable according to predefined positivity criteria. The terminal D1S + D3R pattern was considered positive only when both a terminal S wave in lead I and a terminal R wave in lead III were present within the final 40 ms of the QRS complex with an amplitude ≥0.1 mV. Sensitivity, specificity, positive predictive value (PPV), and area under the curve (AUC) values were calculated using these binary ECG classifications against Qp/Qs ≥ 1.5 and RV/LV ratio > 1 as reference outcomes. Confidence intervals (CI) for AUC values were estimated using 2000 bootstrap resamples. Pairwise comparisons of AUCs were performed using the DeLong test with adjustment for multiple comparisons. Model results were presented as odds ratios (OR), 95% CI, and *p* values. In all analyses, a *p*-value of <0.05 was considered statistically significant.

Agreement between binary ECG parameters and echocardiography-based reference outcomes was assessed using the κ coefficient, while the McNemar test was used to evaluate asymmetry in discordant paired classifications.

## 3. Results

A total of 161 patients were included in the study. Baseline clinical, echocardiographic, and electrocardiographic characteristics according to Qp/Qs status and the presence of RV dilatation are presented in Table 1.

Patients with Qp/Qs ≥ 1.5 had significantly lower left ventricular systolic diameter (*p* = 0.012), larger left atrial diameter (*p* = 0.017), higher PAP (*p* < 0.001), and greater ASD diameter (*p* < 0.001) compared with those with Qp/Qs < 1.5. No significant differences were observed in age (*p* = 0.06), sex (*p* = 0.697), hemoglobin level (*p* = 0.358), left ventricular diastolic diameter (*p* = 0.649), or ejection fraction (*p* = 0.124).

Similarly, patients with RV dilatation had significantly higher PAP (*p* < 0.001), larger ASD diameter (*p* < 0.001), lower left ventricular systolic diameter (*p* = 0.018), and higher left atrial diameter (*p* = 0.019). Age (*p* = 0.312), sex (*p* = 0.506), hemoglobin level (*p* = 0.258), left ventricular diastolic diameter (*p* = 0.164), and ejection fraction (*p* = 0.382) were comparable between groups.

A significant association was observed between RV dilatation and the presence of a hemodynamically significant shunt (Qp/Qs ≥ 1.5) (*p* < 0.001).

Regarding categorical variables, the severity of TR differed significantly between groups for both Qp/Qs (*p* < 0.001) and RV dilatation (*p* < 0.001). Treatment strategy was also significantly associated with both Qp/Qs ≥ 1.5 (*p* < 0.001) and RV dilatation (*p* < 0.001), with a higher proportion of patients undergoing percutaneous closure in these groups.

Among electrocardiographic parameters, RBBB was not significantly associated with either Qp/Qs ≥ 1.5 (*p* = 0.113) or RV dilatation (*p* = 0.063). Right axis deviation was significantly more frequent in patients with RV dilatation (*p* = 0.003), but not in those with Qp/Qs ≥ 1.5 (*p* = 0.303). Increased R-wave amplitude in leads V1–V2 was significantly associated with both Qp/Qs ≥ 1.5 (*p* < 0.001) and RV dilatation (*p* < 0.001). Similarly, the terminal D1S + D3R pattern was significantly more prevalent in patients with Qp/Qs ≥ 1.5 (*p* < 0.001) and in those with RV dilatation (*p* < 0.001).

The EPV values were 14.0 for the Qp/Qs ≥ 1.5 model and 15.2 for the RV dilatation model; both exceeded the commonly used threshold of 10, supporting an acceptable number of outcome events relative to the number of predictors included.

Correlation analyses showed significant positive relationships among right-sided loading parameters. PAP was correlated with ASD diameter using both Pearson and Spearman methods (Pearson r = 0.494, *p* < 0.001; Spearman ρ = 0.631, *p* < 0.001). RV dilatation was strongly correlated with PAP (r_pb = 0.726, *p* < 0.001) and ASD diameter (r_pb = 0.656, *p* < 0.001). TR grade was also positively correlated with PAP (ρ = 0.665, *p* < 0.001), ASD diameter (ρ = 0.419, *p* < 0.001), and RV dilatation (r_rb = 0.503, *p* < 0.001). These findings indicate expected physiological overlap among right-sided loading parameters; therefore, TR grade was not included in the five-variable Firth penalized logistic regression models to reduce redundancy and preserve model stability. Despite these expected physiological correlations, multicollinearity among the predictors included in the final Firth penalized logistic regression models was not substantial, with a maximum VIF of 1.912 and corresponding tolerance values remaining acceptable.

In the reduced Firth penalized logistic regression model evaluating Qp/Qs ≥ 1.5, PAP and ASD diameter were significantly associated with a hemodynamically significant shunt (Table 2). Higher PAP was associated with increased odds of Qp/Qs ≥ 1.5 (OR: 1.14, 95% CI: 1.05–1.26, *p* = 0.002), and larger ASD diameter was also associated with Qp/Qs ≥ 1.5 (OR: 1.44, 95% CI: 1.26–1.70, *p* < 0.001). In contrast, the terminal D1S + D3R pattern was not significantly associated with Qp/Qs ≥ 1.5 after adjustment for age, sex, PAP, and ASD diameter (OR: 0.93, 95% CI: 0.18–4.05, *p* = 0.930). Age (*p* = 0.839) and sex (*p* = 0.649) were also not significantly associated with Qp/Qs ≥ 1.5. The model showed good calibration according to the Hosmer–Lemeshow test (χ^2^ = 7.286, df = 8, *p* = 0.506) and high discriminative performance, with an AUC of 0.965 (95% CI: 0.933–0.997).

In the reduced Firth penalized logistic regression model evaluating RV dilatation, PAP, ASD diameter, and the terminal D1S + D3R pattern were significantly associated with RV dilatation (Table 3). Higher PAP was associated with increased odds of RV dilatation (OR: 1.23, 95% CI: 1.12–1.38, *p* < 0.001), and larger ASD diameter was also significantly associated with RV dilatation (OR: 1.21, 95% CI: 1.07–1.39, *p* = 0.002). The terminal D1S + D3R pattern remained significantly associated with RV dilatation after adjustment for age, sex, PAP, and ASD diameter (OR: 9.90, 95% CI: 2.82–38.20, *p* < 0.001). Age (*p* = 0.119) and sex (*p* = 0.746) were not significantly associated with RV dilatation. The model showed good calibration according to the Hosmer–Lemeshow test (χ^2^ = 6.292, df = 8, *p* = 0.615) and high discriminative performance, with an AUC of 0.980 (95% CI: 0.962–0.999).

The diagnostic performance of individual electrocardiographic parameters is summarized in Table 4. In the ROC analyses, 95% confidence intervals for AUC values were estimated using 2000 bootstrap resamples, and pairwise comparisons of AUC values were performed using the DeLong test. For detecting Qp/Qs ≥ 1.5, the terminal D1S + D3R pattern demonstrated acceptable diagnostic performance, with an AUC of 0.798 (95% CI: 0.732–0.863), sensitivity of 72.9%, specificity of 86.7%, and PPV of 83.6%. For detecting RV dilatation, the terminal D1S + D3R pattern showed good diagnostic performance, with an AUC of 0.881 (95% CI: 0.831–0.932), sensitivity of 86.8%, specificity of 89.4%, and PPV of 88.0%. Other ECG parameters showed lower overall discriminative performance. In pairwise DeLong comparisons, the AUC of the terminal D1S + D3R pattern was significantly higher than that of RBBB, right axis deviation, and R-wave positivity in V1–V2.

Agreement analyses are presented in Table 5. These analyses were performed to evaluate the concordance between binary ECG parameters and echocardiography-based reference outcomes, rather than to define diagnostic cut-off values. κ analysis was used to assess the degree of agreement, whereas the McNemar test was used to evaluate asymmetry in discordant paired classifications. The terminal D1S + D3R pattern showed moderate agreement with Qp/Qs ≥ 1.5 (κ = 0.598) and substantial agreement with RV dilatation (κ = 0.763), with non-significant McNemar test results (*p* = 0.136 and *p* = 1.000, respectively). These findings indicate that the terminal D1S + D3R pattern had the closest concordance with echocardiography-defined RV dilatation among the evaluated ECG parameters.

In contrast, other electrocardiographic parameters demonstrated weak agreement with echocardiographic findings, including R-wave positivity in leads V1–V2 (κ = 0.293 and 0.238), right axis deviation (κ = 0.063 and 0.154), and RBBB (κ = 0.130 and 0.145), all with significant McNemar test results (*p* < 0.001).

## 4. Discussion

In this study, we primarily evaluated the diagnostic value of the terminal D1S + D3R pattern in relation to right heart overload in patients with secundum ASD. In the multivariable Firth penalized logistic regression model, PAP, ASD diameter, and the terminal D1S + D3R pattern were significantly associated with RV dilatation. The terminal D1S + D3R pattern remained independently associated with RV dilatation (OR: 9.90, 95% CI: 2.82–38.20, *p* < 0.001). Although the effect size was large, the wide CI suggests possible estimate instability; therefore, the precise magnitude of the OR should be interpreted cautiously. PAP (OR: 1.23, 95% CI: 1.12–1.38, *p* < 0.001) and ASD diameter (OR: 1.21, 95% CI: 1.07–1.39, *p* = 0.002) were also independently associated with RV dilatation.

In contrast, the terminal D1S + D3R pattern was not significantly associated with Qp/Qs ≥ 1.5 in the multivariable Firth penalized logistic regression model (OR: 0.93, 95% CI: 0.18–4.05, *p* = 0.930), whereas PAP and ASD diameter remained significantly associated with Qp/Qs ≥ 1.5. Although the terminal D1S + D3R pattern showed diagnostic performance for Qp/Qs ≥ 1.5 in individual ROC analysis, the absence of an independent association after adjustment for PAP and ASD diameter suggests that the relationship between terminal D1S + D3R and shunt burden may be mediated through associated structural remodeling rather than hemodynamic load per se. Therefore, the terminal D1S + D3R pattern should be interpreted as an ECG marker more closely related to RV dilatation than to Qp/Qs-defined shunt magnitude.

These findings suggest that while hemodynamic burden, as reflected by Qp/Qs, is primarily associated with PAP and defect size, the terminal D1S + D3R pattern may more closely reflect structural remodeling of the right ventricle.

Patients with ASD may remain asymptomatic for a long time; however, some patients may present with fatigue, arrhythmia, or exercise intolerance. Volume overload in the right heart eventually leads to increased pulmonary pressure, and the emergence of these hemodynamic effects becomes more apparent. Current guidelines define hemodynamic effects as a Qp/Qs ratio ≥ 1.5 and right-sided cardiac chamber enlargement. These hemodynamic findings, together with a history of paradoxical embolism, are considered within the scope of indications for intervention [7].

The emergence of right heart overload and the development of PAH in patients with ASD are associated with increased mortality and morbidity [8,9]. It has also been shown to cause reductions in patients’ exercise capacity and decreases in quality of life [10,11]. In cases where right heart failure develops, it can lead to an increase in the hospitalization rate [12].

Echocardiography remains essential for diagnosis, follow-up, and assessment of right heart involvement in patients with ASD. However, simple and inexpensive ECG markers may provide complementary clinical information and increase awareness of possible RV involvement before definitive echocardiographic assessment.

In previous studies, ECG evaluation was shown to be useful in predicting the development of PAH in patients with ASD secundum [13,14,15]. Right axis deviation and the development of incomplete RBBB are common ECG findings [16].

In the pediatric study, RBBB correlated significantly with higher Qp/Qs values and decreased markedly after surgical closure, indicating that RBBB is a load-dependent and dynamic marker in children [17]. In our adult cohort, RBBB was not significantly associated with either Qp/Qs ≥ 1.5 or RV dilatation in descriptive comparisons and showed limited diagnostic performance in ROC analysis. By contrast, the terminal D1S + D3R pattern showed stronger diagnostic performance and closer concordance with RV dilatation, suggesting that terminal vector changes may better capture ECG alterations accompanying right-sided structural enlargement in adult patients with secundum ASD. However, this interpretation remains hypothesis-generating and should not be considered direct evidence of electrical remodeling.

Previous pediatric studies demonstrating that the rSR′ pattern correlates with shunt magnitude and right ventricular volume loading provide important physiological support for the concept that RV overload can manifest through characteristic surface ECG findings [18]. In line with this physiological background, our adult secundum ASD cohort showed that the terminal D1S + D3R pattern is significantly associated with structural (RV/LV > 1) indicators of RV volume overload, whereas no significant association was observed with Qp/Qs ≥ 1.5 in multivariable analysis. Taken together, these findings suggest that the terminal D1S + D3R pattern is associated with ECG terminal vector changes consistent with delayed RV activation, likely secondary to chronic RV volume loading and structural remodeling in adult patients with secundum ASD. Nevertheless, the present study demonstrates an association rather than a direct causal or pathophysiological relationship, and further prospective studies are needed to clarify the electrophysiological basis of this ECG pattern. However, as in the pediatric literature, such ECG findings should be interpreted as guiding signals of potential RV overload rather than replacements for echocardiographic assessment, which remains essential for diagnostic confirmation and management decisions.

In patients diagnosed with acute pulmonary embolism, the presence of terminal D1S + D3R was found to be an independent predictor of RV dilatation [19]. In our study, we also evaluated the relationship between the detection of terminal D1S + D3R in patients diagnosed with ASD and signs of right heart overload. Compared with conventional electrocardiographic markers such as right axis deviation, RBBB, and V1–V2 R-wave positivity, the terminal D1S + D3R pattern showed higher diagnostic performance and closer concordance with RV dilatation in the present cohort. In our cohort, the positivity rate of D1S + D3R reached 88% in the presence of RV dilatation, whereas right axis deviation, RBBB, and V1–V2R positivity were observed in only 17%, 39%, and 16% of patients, respectively. This difference may be related to the ability of terminal-phase vector changes to capture ECG alterations accompanying RV dilatation more effectively than conventional markers. However, because the present study was observational, these findings should be interpreted as an association between the D1S + D3R pattern and RV dilatation rather than evidence of causality or a direct pathophysiological relationship. The discrepancy between our findings and those reported in patients with pulmonary embolism can be explained by distinct pathophysiological mechanisms. Pulmonary embolism produces an acute increase in right ventricular afterload, leading to abrupt dilation and sharp vector changes, whereas secundum ASD leads to chronic volume overload, with slower remodeling and a different electrophysiological signature. Consistent with this interpretation, the terminal D1S + D3R pattern was observed more frequently in patients with RV dilatation than in those without RV dilatation.

The potential clinical role of the terminal D1S + D3R pattern may be considered as an inexpensive and readily available screening adjunct that can complement echocardiographic evaluation. Given its association with RV dilatation, this ECG finding may increase clinical awareness of possible right-sided structural involvement in patients with secundum ASD. However, the terminal D1S + D3R pattern should not be interpreted as a stand-alone diagnostic, triage, or treatment-guiding tool. Echocardiographic assessment remains essential for confirming RV dilatation, evaluating shunt burden, and guiding clinical management decisions.

This study has several limitations. First, its single-center and retrospective design may limit the generalizability of the findings and introduce potential selection bias. In addition, referral patterns to a tertiary care center, as well as clinical decision-making regarding medical follow-up versus percutaneous intervention, may have influenced the characteristics of the study population.

Second, hemodynamic significance was assessed using echocardiography-derived Qp/Qs measurements, as invasive right heart catheterization data were not available for all patients. Although echocardiographic Qp/Qs assessment is widely used in clinical practice, it is subject to operator-dependent variability and may result in potential misclassification, particularly in cases with borderline values. Therefore, the results should be interpreted in light of this methodological limitation.

Furthermore, long-term prognostic follow-up data were not available, which limits the ability to objectively assess the clinical course and long-term consequences of right heart loading. Although the terminal D1S + D3R pattern may be consistent with delayed RV activation and terminal vector changes, the present study did not directly assess electrophysiological remodeling; therefore, this interpretation should be considered hypothesis-generating rather than definitive. In addition, all ECG parameters were evaluated by two cardiologists blinded to clinical and echocardiographic data through a consensus-based assessment; however, formal interobserver and intraobserver reproducibility analyses were not available. Thus, potential observer dependency in the manual assessment of ECG parameters, particularly the terminal D1S + D3R pattern, should be acknowledged. Finally, the terminal D1S + D3R pattern was evaluated as a binary ECG variable. Continuous measurements such as terminal S-wave amplitude in lead I, terminal R-wave amplitude in lead III, or a summed terminal amplitude index were not analyzed; therefore, the potential dose–response relationship between the magnitude of terminal vector changes and RV dilatation could not be assessed and should be addressed in future studies.

## 5. Conclusions

In adults with secundum ASD, a positive terminal D1S + D3R ECG pattern, defined as the coexistence of a terminal S wave in lead I and a terminal R wave in lead III, is independently associated with RV dilatation and demonstrates good discriminatory performance for identifying structural right heart remodeling. Although this ECG pattern should not replace echocardiographic assessment, its presence may serve as a practical adjunctive screening marker to increase clinical awareness of possible RV dilatation.

## Figures and Tables

**Table 1:** Baseline Clinical, Electrocardiography, and Echocardiographic Characteristics According to Qp/Qs and RV Dilatation.

Parameter		Qp/Qs < 1.5(*n* = 75)		Qp/Qs ≥ 1.5 (*n* = 70)		*p*	RV Dilatation Absent (*n* = 85)		RV Dilatation Present (*n* = 76)		*p*
		Median	IQR	Median	IQR		Median	IQR	Median	IQR	
**Age**		32	24–51	41	31–53	0.06	37	25–50	40	29–52.5	0.312
Hb (mg/dL)		13.5	12.7-	13.8	12.7–15.1	0.358	13.5	12.7–14.5	13.85	12.7–15.2	0.258
EF (%)		65	60–65	65	60–65	0.124	65	60–65	65	60–65	0.382
LVDD (mm)		44	41–46	43	41–46	0.649	44	41–46	42.5	40.5–45.5	0.164
LVSD (mm)		25	24–29	24	22–27	0.012	25	24–29	24	21–27	0.018
LA diameter (mm)		32	29–36	34	31–37	0.017	32	30–36	34	32–37	0.019
PAP (mmHg)		26	22–31	40	34–45	<0.001	26	23–31	40.5	37.5–45	<0.001
ASD diameter (mm)		4	3–6	16	12–20	<0.001	4	3–8	16	12–21	<0.001
**Parameter**		Number (*n*)	(%)	Number (*n*)	(%)	*p*	Number (*n*)	(%)	Number (*n*)	(%)	*p*
Gender	Female	47	50.00%	47	50.00%	0.697	54	50.47%	53	49.53%	0.506
	Male	28	54.90%	23	45.10%		31	57.41%	23	42.59%	
TR	Absent	28	73.68%	10	26.32%	<0.001	33	84.62%	6	15.38%	<0.001
	Stage 1	41	52.56%	37	47.44%		46	53.49%	40	46.51%	
	Stage 2	5	21.74%	18	78.26%		5	17.24%	24	82.76%	
	Stage 3	0	0.00%	5	100.00%		0	0.00%	6	100.00%	
Qp/Qs	Absent						70	93.33%	5	6.67%	<0.001
	Present						14	20.00%	56	80.00%	
RV Dilatation	Absent	70	83.33%	14	16.67%	<0.001					
	Present	5	8.20%	56	91.80%						
RBBB	Absent	61	55.96%	48	44.04%	0.113	68	57.63%	50	42.37%	0.063
	Present	14	38.89%	22	61.11%		17	39.53%	26	60.47%	
Right Axis	Absent	71	53.38%	62	46.62%	0.303	83	56.85%	63	43.15%	0.003
	Present	4	33.33%	8	66.67%		2	13.33%	13	86.67%	
V1–V2R	Absent	74	60.16%	49	39.84%	<0.001	81	59.56%	55	40.44%	<0.001
	Present	1	4.55%	21	95.45%		4	16.00%	21	84.00%	
D1S + D3R	Absent	65	77.38%	19	22.62%	<0.001	76	88.37%	10	11.63%	<0.001
	Present	10	16.39%	51	83.61%		9	12.00%	66	88.00%	
Treatment	Medical	68	95.77%	4	4.23%	<0.001	70	98.59%	1	1.41%	<0.001
	Operation	7	9.46%	67	90.54%		15	16.67%	75	83.33%	

**Abbreviations:** Hb; Hemoglobin, EF; Ejection Fraction, LVDD; Left Ventricular Diastolic Diameter, LVSD; Left Ventricular Systolic Diameter, LA; Left Atrium, PAP; Pulmonary Artery Pressure, ASD; Atrial Septal Defect, TR; Tricuspid Regurgitation, RV; Right Ventricle, RBBB; Right Bundle Branch Block, Qp/Qs; Pulmonary-to-Systemic Flow Ratio, V1–V2R; R wave in V1–V2 leads, D1S + D3R; Terminal S wave in lead I and terminal R wave in lead III.

**Table 2:** Firth Penalized Logistic Regression Analysis for Qp/Qs ≥ 1.5.

Variable	OR	95% CI	*p* Value
Age	1.00	0.96–1.03	0.839
Sex	0.75	0.22–2.57	0.649
PAP	1.14	1.05–1.26	0.002
Terminal D1S + D3R pattern	0.93	0.18–4.05	0.930
ASD diameter	1.44	1.26–1.70	<0.001

**Abbreviations:** OR; Odds Ratio, CI; Confidence Interval, ASD; Atrial septal defect, Qp/Qs; Pulmonary-to-Systemic Flow Ratio, D1S + D3R; Terminal S wave in lead I and terminal R wave in lead III, PAP; pulmonary artery pressure. Footnote: Multivariable Firth penalized logistic regression was performed to reduce potential sparse data bias and quasi-complete separation. The dependent variable was Qp/Qs ≥ 1.5. The model included 145 complete cases.

**Table 3:** Firth Penalized Logistic Regression Analysis for RV Dilatation.

Variable	OR	95% CI	*p* Value
Age	0.96	0.92–1.01	0.119
Sex	0.80	0.20–3.23	0.746
PAP	1.23	1.12–1.38	<0.001
Terminal D1S + D3R pattern	9.90	2.82–38.20	<0.001
ASD diameter	1.21	1.07–1.39	0.002

**Abbreviations:** OR; Odds Ratio, CI; Confidence Interval, ASD; Atrial septal defect, RV; Right Ventricle, D1S + D3R; Terminal S wave in lead I and terminal R wave in lead III, PAP; pulmonary artery pressure. Footnote: Multivariable Firth penalized logistic regression was performed to reduce potential sparse data bias and quasi-complete separation. The dependent variable was RV dilatation. The model included 161 complete cases.

**Table 4:** Diagnostic Performance of Individual ECG Parameters for Qp/Qs ≥ 1.5 and RV Dilatation.

Parameters	Qp/Qs ≥ 1.5 AUC	95% CI	Sensitivity	Specificity	PPV	RV Dilatation AUC	95% CI	Sensitivity	Specificity	PPV
Terminal D1S + D3R pattern	0.798	0.732–0.863	72.9%	86.7%	83.6%	0.881	0.831–0.932	86.8%	89.4%	88.0%
RBBB	0.564	0.493–0.634	31.4%	81.3%	61.1%	0.571	0.502–0.640	34.2%	80.0%	60.5%
Right axis deviation	0.530	0.485–0.576	11.4%	94.7%	66.7%	0.574	0.528–0.619	17.1%	97.6%	86.7%
R-wave positivity in V1–V2	0.643	0.588–0.699	30.0%	98.7%	95.5%	0.615	0.559–0.670	27.6%	95.3%	84.0%

**Abbreviations:** AUC, area under the curve; CI, confidence interval; PPV, positive predictive value; ECG, electrocardiography; RBBB, right bundle branch block; Qp/Qs, pulmonary-to-systemic flow ratio; RV, right ventricle; D1S + D3R, terminal S wave in lead I and terminal R wave in lead III. Footnote: Diagnostic performance analyses were performed for individual binary ECG parameters based on predefined positivity criteria. For the terminal D1S + D3R pattern, positivity required the coexistence of a terminal S wave in lead I and a terminal R wave in lead III within the final 40 ms of the QRS complex with an amplitude ≥0.1 mV. Sensitivity, specificity, PPV, and AUC values were calculated against Qp/Qs ≥ 1.5 and RV/LV ratio >1 as reference outcomes. These results should be interpreted separately from the multivariable Firth penalized regression models presented in Table 2 and Table 3.

**Table 5:** Concordance of ECG Parameters with Qp/Qs and RV Dilatation (McNemar Test and κ Analysis).

Parameters	Qp/Qs ≥ 1.5			RV Dilatation		
	McNemar *p*	κ	Overall Agreement	McNemar *p*	κ	Overall Agreement
Terminal D1S + D3R pattern	0.136	0.598	0.8	1	0.763	0.881
V1–V2 R-wave positivity	<0.001	0.293	0.655	<0.001	0.238	0.633
Right Axis	<0.001	0.063	0.544	<0.001	0.154	0.596
RBBB	<0.001	0.13	0.572	<0.001	0.145	0.583

**Abbreviations:** RBBB; Right Bundle Branch Block, Qp/Qs; Pulmonary-to-Systemic Flow Ratio, D1S + D3R; Terminal S wave in lead I and terminal R wave in lead III, RV; Right Ventricle. Footnote: κ values indicate agreement between each binary ECG parameter and the corresponding echocardiography-based outcome, whereas the McNemar test evaluates asymmetry in discordant paired classifications.

## Data Availability

The data sets created and/or analyzed during the current study are not publicly available because they contain patient information, but data supporting the findings of this study can be obtained from the corresponding author (RA) upon reasonable request.

## Abbreviations

The following abbreviations are used in this manuscript:

ASD	Atrial septal defect
AUC	Area under the curve
CHD	Congenital heart disease
CI	Confidence interval
ECG	Electrocardiography
EPV	Events per variable
LV	Left ventricle
OR	Odds ratio
PAH	Pulmonary arterial hypertension
PAP	Pulmonary artery pressure
PPV	Positive predictive value
Qp/Qs	Pulmonary-to-systemic flow ratio
RBBB	Right bundle branch block
RHC	Right heart catheterization
ROC	Receiver operating characteristic
RV	Right ventricle
RV/LV	Right ventricle/left ventricle ratio
TEE	Transesophageal echocardiography
TR	Tricuspid regurgitation
V1–V2R	R-wave positivity in leads V1–V2
VIF	Variance inflation factor
